# Magnetic Resonance Imaging-Guided Online Adaptive Lattice Stereotactic Body Radiotherapy in Voluminous Liver Metastasis: Two Case Reports

**DOI:** 10.7759/cureus.23980

**Published:** 2022-04-09

**Authors:** Neris Dincer, Gamze Ugurluer, Latif Korkmaz, Anatolia Serkizyan, Banu Atalar, Gorkem Gungor, Enis Ozyar

**Affiliations:** 1 Radiation Oncology, Acibadem University, Istanbul, TUR; 2 Radiation Oncology, Acibadem Maslak Hospital, Istanbul, TUR; 3 Radiation Oncology, Acibadem Mehmet Ali Aydinlar University School of Medicine, Istanbul, TUR; 4 Radiation Oncology, Acibadem Hospital, Istanbul, TUR

**Keywords:** lattice, mr-linac, sbrt, inoperable, liver metastasis

## Abstract

Lattice Radiotherapy (LRT) is a technique in which heterogeneous doses are delivered to the target so large tumors can have optimal doses of radiation without compromising healthy tissue sparing. To date, case reports and case series documented its application for bulky tumors mainly in the pelvic region. LRT not only provides dosimetric advantages but also promotes tumor control by triggering some radiobiological and immunological pathways. We report two cases of giant liver metastases for whom other treatment options were not suitable. We treated both patients with Magnetic Resonance Image-Guided Radiotherapy (MRgRT) with online adaptive LRT (OALRT) technique. Adaptive plans were generated before each fraction. Tumors were observed to have regressed interfractionally so the location and number of spheres were adapted to tumor size and daily anatomy of the surrounding organs at risk (OAR). Both patients had good treatment compliance without any Grade 3+ side effects. They are both under follow-up and report improvement. By reporting the first application of OALRT by using MRgRT in liver metastases, we show that MRgRT is a promising modality for LRT technique with better target and OAR visualization as well as online adaptive planning before each fraction according to the daily anatomy of the patient.

## Introduction

Treatment of voluminous tumors represents a challenge to radiation oncologists as delivery of high doses to the target lesions bring along the risk of not securing healthy tissue sparing. Spatially Fractionated Radiation Therapy (SFRT) modality was introduced in order the overcome this problem with the idea that the tumor could be irradiated with a heterogeneous dose distribution [1]. SFTR not only offers better healthy organ protection but also has radiobiological and immunomodulatory mechanisms [2]. Lattice radiotherapy (LTR) is derived from this concept with three-dimensional dose delivery [3]. Valley and vertex volumes are defined in the tumor, and they are delivered in lower and higher doses respectively [2]. LRT is a promising technique in the treatment of bulky tumors.

Herein we report the management of two cases with bulky liver metastases presenting to our clinic. Both patients were not amenable to surgery due to volumes of metastatic lesions. We decided to treat the patient with online adaptive LRT (OALRT) with the aim of preserving the surrounding tissue and benefiting from heterogeneous dose distribution. We have treated both patients by using MRgRT, which enabled better soft-tissue visibility, adaptive planning, and online continuous cine tracking of tumors during the treatment. To our knowledge, this report is the first on LRT using MRgRT in liver metastases.

## Case presentation

Case 1

A 59-year-old female with a non-significant past medical history and family history was diagnosed with squamous cell cancer of the anal canal in 2018 and staged as T2N0M0. Positron emission tomography-CT (PET-CT) revealed a distal rectum tumor with pathological fludeoxyglucose (FDG) uptake in the lymph nodes at the level of the right presacral and lumbal 3 vertebrae. Chemoradiotherapy was offered to the patient, but she refused the treatment. In March 2020, she received 20 Gy in 5 fractions to the pelvic area as a palliative treatment. In the follow-ups, the tumor responded well to treatment but a metastatic lesion in the liver was detected. The patient declined to take chemotherapy and went with alternative medicine. In October 2020, liver metastases were found to have progressed. She did not receive any treatment. In May 2021, she received 36 Gy to pelvic lymph nodes and 50 Gy to the primary tumor bed in 20 fractions with a simultaneously integrated boost technique. Computed tomography was performed in July 2021, and it revealed giant metastatic liver lesions in segments 4B and 6. At that time, her aspartate aminotransferase (AST) was 33 IU/L, and alanine aminotransferase (ALT) was 13 IU/L. Her Gamma-glutamyltransferase was above normal limits and it was 147 IU/L. Chemotherapy was refused by the patient. She was referred to our clinic for stereotactic body radiotherapy (SBRT).

Case 2

A 70-year-old male patient underwent colonic resection and liver metastasectomy due to rectosigmoid adenocarcinoma in March 2016. Pathological staging was consistent with T3N1M1. He received adjuvant chemotherapy. Between 2019 and 2021, he received chemotherapies and underwent radiofrequency ablation and transarterial chemoembolization (TACE) due to the recurrence of his liver metastases. In 2021, contrasted abdominal CT revealed a large mass in the right lobe of the liver as well as progression in the mass to which TACE was performed. Due to the pandemic, the patient delayed the treatment and he presented to our clinic in October 2021. His last PET-CT report of August 2021 showed up to eight nodules with FDG uptake, and it was consistent with progression compared with his previous PET-CT results. The patient's ALT was 36 IU/L and AST was slightly above the reference range at 50 IU/L.

We decided to go with SBRT with lattice treatment planning in MRIdian Linac (ViewRay Inc, Mountain View, USA).

SBRT-Lattice Treatment

We decided to perform SBRT using MRIdian Linac for both patients given the lesions were giant masses located in the liver. The patients were scanned by using a planning CT for electron density map, diagnostic MRI, and Truefisp sequence of MRIdian Linac. Sequences were taken under end-inspirium breath-hold. All the available images were fused for treatment planning.

Gross Tumor Volume (GTV) was delineated as the primary tumor. Planning Target Volume (PTV) was extended 3 mm in all directions from GTV. OARs were determined as: esophagus, stomach, spinal cord, superior mesenteric artery, portal vein, pancreas, lungs, liver, bowels, right and left kidney, heart, duodenum, celiac artery, and aorta. A total dose of 30-50 Gy in 5 fractions (6-10 Gy/fraction) was prescribed to the metastatic lesion as valley and lattice structures as the peak. A step and shoot Intensity Modulated Radiotherapy (IMRT) treatment plan was generated with 18 and 23 fields in a total of 119 and 123 segments, respectively, with a single isocenter and 6 megavolt (MV) flattening filter free (FFF) photon energy.

For case 1, the dose-volume histogram (DVH) revealed that the dose to 700 ccs of the liver was 10.7 Gy; V33 and V36 of the stomach were 0, and the mean dose to the right kidney was 11.04 Gy while the mean dose to left kidney was 11.4 Gy. The patient's duodenum could not be separately contoured because of the patient's anatomical variations and V36 of bowels was 0.04 cc and V33 of bowels was 1.29 cc. The GTVs were heterogeneous, so the homogeneity index (HI) was calculated for the whole PTV, and it was 0.65, and the conformity index (CI) was 1.03.

For case 2, the dose to 700 ccs of the liver was 28.49 Gy; V33 and V36 of the stomach were 0, and the mean dose to the right kidney was 9.1 Gy while the mean dose to the left kidney was 3.37 Gy. V33 and V36 of the duodenum were 1.03 and 0.23 cc respectively and V33 and V36 of bowels were both 0. HI was calculated as previously mentioned and it was 0.58. CI was 1.33.

**Figure 1:**
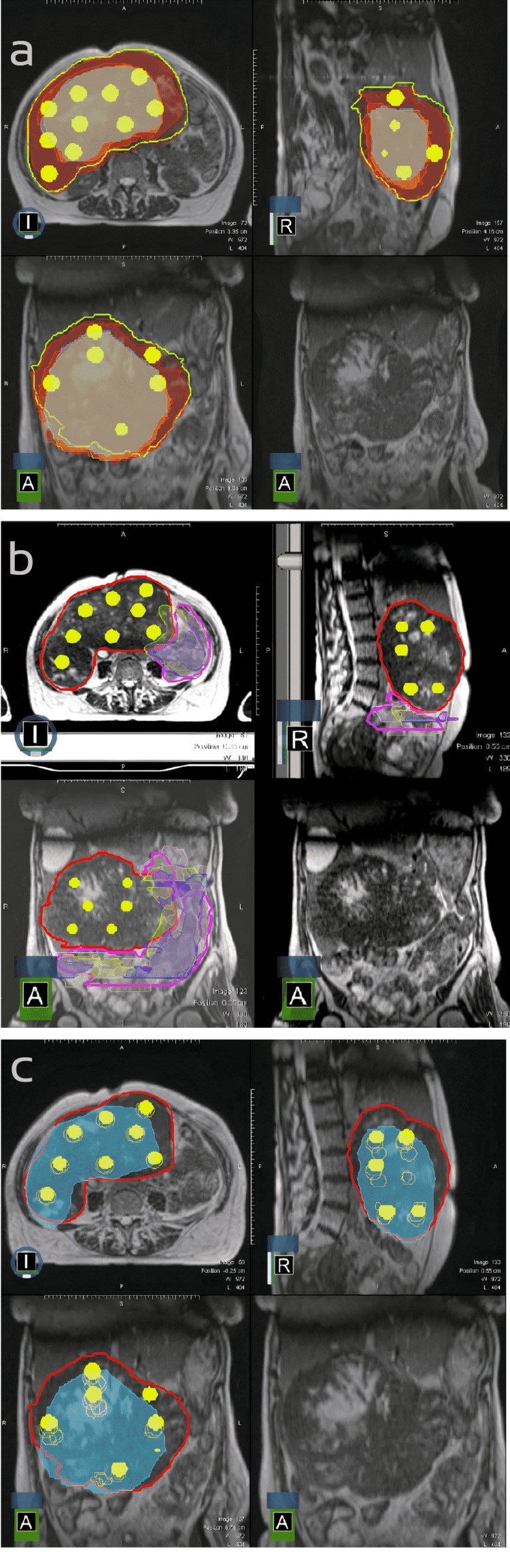
GTV and the location of spheres in the original and reoptimized plans Figure 1a: Yellow line represents the original GTV contour and the yellow spheres represent peak areas. The figure shows the shrinkage of the GTV over the fractions. Note that spheres would be located within OARs if adaptive planning was not performed Figure 1b: The red line is the original GTV and the yellow spheres are the peak areas. The changes in the bowel contour over the fractions are shown with pink, blue, and yellow lines. Figure 1c: The original GTV contour and blue area represent the volumes in the following fractions. Spheres that are located outside can be seen and note that they were deleted during adaptive planning. Yellow-colored filled spheres represent the original peak areas. Empty spheres represent the peak areas in the following fractions. Note that their location changed in accordance with the daily anatomy. GTV: Gross Tumor Volume; OAR: Organs at Risk

An adaptive online plan was generated during all fractions (5 of 5 fractions) for both patients. The lattice spheres were relocated (or erased) if needed due to gross tumor volume shrinkage and proximity to OARs during each fraction. The plan of the original and reoptimized target volumes and spheres as well as the close proximity between GTV and OARs for case 1 are demonstrated in Figures 1a and 1b, respectively. Figure 1a also demonstrates that the normal tissues would be irradiated with peak doses if the adaptive plan was not performed. These two figures are a striking example of how daily anatomy changes for target tissues and OARs and how irradiation of normal tissue would be unavoidable if adaptive planning was not performed. The change in GTV contour, as well as the location of spheres over the fractions, are shown in Figure 1c.

**Figure 2:**
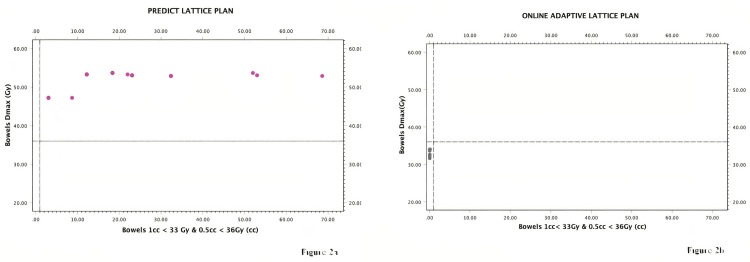
Graphical illustrations of bowel doses Figure 2a shows the bowel doses in predicted plans and Figure 2b shows the bowel doses that we obtained via online adaptive planning. Note that bowel dose restrictions would be violated without adaptive planning. Gy: Gray

**Figure 3:**
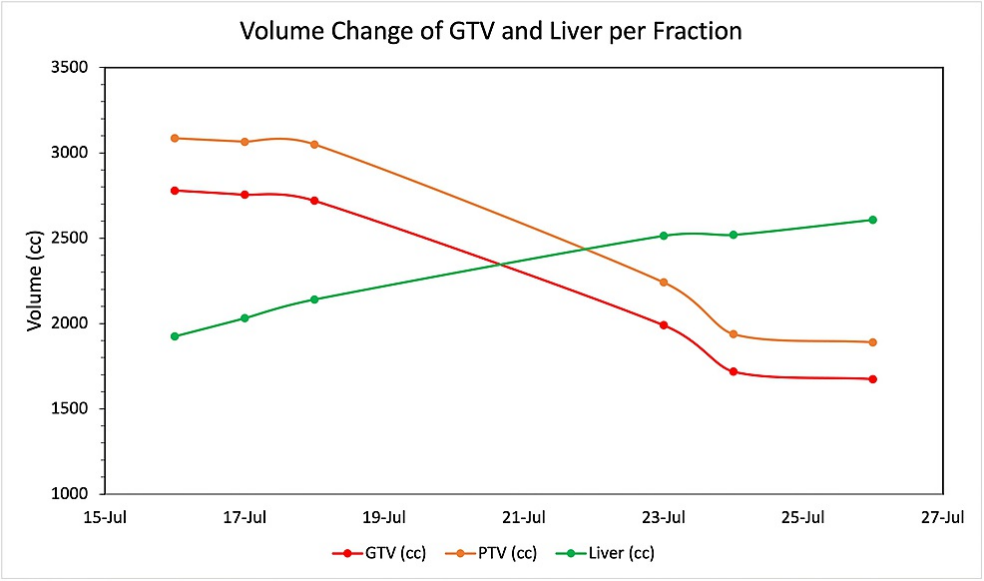
Change in GTV, PTV and liver volume The graph illustrates the change in the GTV volume, PTV volume as well as liver volume over the fractions. GTV: Gross Tumor Volume; PTV: Planning Target Volume

Figures 2a-2b are graphical illustrations of bowel doses if adaptive treatment was not applied for each fraction and doses received by bowel with adaptive planning. Figure 3 demonstrates the change in GTV, PTV, and liver volumes over the fractions.

Both patients tolerated the treatment well without any acute Grade 3-4 toxicity according to CTCAE (Common Terminology Criteria for Adverse Events) v4. For Case 1, the mass was visibly regressed at the end of 5 fractions. PET-CT scan in the third month revealed that the mass showed near-complete regression. The patient accepted the chemotherapy and she will be evaluated for surgery. Case 2 showed a mild increase in liver function tests in the second fraction, but it did not persist. One month after the treatment, an abdominal CT revealed a regression in the dimension of GTV (pre-treatment GTV 494 cc to post-treatment GTV 230 cc).

## Discussion

Spatially Fractionated Radiation Therapy differs from the standard radiotherapy approach by delivering in homogeneous doses to the target tissue. It has been decades since this concept was defined and the primary idea behind it was to palliate symptoms caused by bulky tumors by giving less dose to the areas close to OAR while still reaching a high biologically effective dose at the tumor [4]. The historical application of SFRT was in the orthovoltage era and it was called GRIDradiotherapy. A heterogeneous dose was delivered by a perforated scan and blocked areas [1]. Although the GRID approach lost its popularity with the usage of megavoltage X-rays, it was later adapted to the technology of that time and gave promising results not only in palliative but also in definitive settings without severe toxicity [1, 5].

In GRID radiotherapy, the tumor is divided into valley and peak areas. Valleys, also called cold spots, are areas that are receiving the lower radiation dose, whereas peaks, also called hot spots, are the areas where the higher dose is prescribed [2]. In the result of these, a valley-to-peak ratio can be calculated to quantify the dose heterogeneity within the tumor [6]. One can deduce that a steep dose reduction is necessary at the intersection points which can be disrupted by radiation leakage in between the leaves [6].

It was not until 2010 that we were introduced to LRT which can be defined as a three-dimensional extension of GRID radiotherapy [7]. LRT provided higher doses to higher volumes while better sparing OAR [8] and better targeting overcame the previously mentioned radiation leakage problem. In the three-dimensional setting, spheres are formed in the tumor, and these are the high-dose areas.

The dosimetric advantage yielded by LRT not only demonstrates technical advantages but also helps disease control by triggering various radiobiological and immunogenic mechanisms [5]. The radiation-induced bystander effect is the biological outcome observed in unirradiated contiguous cells as if they were exposed to radiation and in vitro studies demonstrated that it occurs through intercellular signaling [9]. In LRT, the bystander effect is not only observed in contiguous unirradiated cells but also in the valley region resulting in enhanced radiation response throughout the target [5]. Radiation also induces pro-apoptotic signals in endothelial cells, thus microvasculature of the tumor is disrupted [6]. This effect is more pronounced in high doses [10] of radiation, so LRT is effective in mitigating the supply of tumor cells [11]. Immunomodulatory effects of radiotherapy are also enhanced with LRT owing to higher radiation doses that can be delivered with this technique [12].

Indications of LRT are expected to widen in the future with its use not only limited to bulky tumors since we can also benefit from its radiobiological and immune-modulator effects [7]. It is estimated that over 150 patients with bulky tumors received LRT [7]. To date, our knowledge is limited to several case reports and case series. There is no consensus on peak-valley doses and the optimal dose should be individualized according to tumor biology, tumor size, and surrounding organs. Blanco Suarez et al. report promising results in terms of local control with their experience of over 20 patients [13]**.** In their retrospective analysis of 10 non-small cell lung cancer patients, Amendola et al. report a mean tumor size reduction of 52% with no grade 2-3 radiation pneumonitis, hence they declared that LRT is a safe technique to apply cytotoxic radio-therapeutical doses [14]. They applied cone-beam CT (CBCT) before every fraction, and they performed an adaptive plan for rapid and significant decrements in tumor volume. Recently, Pollack et al. published the results of their phase 1 trial in which they applied the Lattice Extreme Ablatice Dose (LEAD) technique to MRI-defined GTV volumes. The LEAD technique let them deliver doses higher than 80 Gy with no gastrointestinal side effects above Grade 2 and Grade 2+ genitourinary side effects that are transient [15].

Duraseti et al. reported a case series of 11 patients with >12 cm tumors. They prescribed a dose of 2000 cGy to the entire tumor and 6670 cGy to peak regions. The tumors were of different histologies from various sites. Dose constraints could be achieved in all patients except one in whom the tumor was in close proximity to skin and all plans achieved adequate coverage. They concluded that the planning can be successfully performed for bulky tumors [16]. In their consecutive phase 1 trial, 22 patients were enrolled and the endpoints were quality of life and acute toxicity. Position verification was performed with CBCT, orthovoltage Kv imaging as well as fluoroscopic imaging for thoracic tumors. The Median GTV volume was 579.2 cc. One patient had grade 3+ toxicity (urosepsis) and one had grade 2 pneumonitis. All lesions were observed to have shrunk. The conclusion is that Lattice offers a treatment option for large tumors with acceptable toxicity and no deterioration in the quality of life [17].

## Conclusions

Herein, to our knowledge, we present the first documented case of LRT application in an MR-Linac device with adaptive planning in each fraction. MRgRT allowed us to visualize the tumor with the advantages of better soft tissue discrimination in MRI with daily adaptive planning and modulation of sphere location and sizes by seeing the regression in the target volume. By means of this, the spheres that would be in proximity to surrounding tissue due to tumor shrinkage were deleted and high doses to vulnerable tissues were avoided. The patients tolerated the treatment well with visible tumor regression. LRT is a promising technique in bulky tumors, and it enables the delivery of higher doses without compromising on dose constraints. MRgRT can be safely and effectively used for LRT.

## References

[1] Yan W, Khan MK, Wu X (2020). Spatially fractionated radiation therapy: history, present and the future. Clin Transl Radiat Oncol.

[2] Pellizzon AC (2020). Lattice radiation therapy - its concept and impact in the immunomodulation cancer treatment era. Rev Assoc Med Bras (1992).

[3] Amendola BE, Perez NC, Wu X, Blanco Suarez JM, Lu JJ, Amendola M (2018). Improved outcome of treating locally advanced lung cancer with the use of lattice radiotherapy (LRT): a case report. Clin Transl Radiat Oncol.

[4] Mohiuddin M, Fujita M, Regine WF, Megooni AS, Ibbott GS, Ahmed MM (1999). High-dose spatially-fractionated radiation (GRID): a new paradigm in the management of advanced cancers. Int J Radiat Oncol Biol Phys.

[5] Billena C, Khan AJ (2019). A current review of spatial fractionation: back to the future?. Int J Radiat Oncol Biol Phys.

[6] Ferini G, Valenti V, Tripoli A (2021). Lattice or oxygen-guided radiotherapy: what if they converge? Possible future directions in the era of immunotherapy. Cancers (Basel).

[7] Wu X, Perez NC, Zheng Y (2020). The technical and clinical implementation of LATTICE radiation therapy (LRT). Radiat Res.

[8] Wu X, Ahmet MM, Pollack A. (2009). On modern technical approaches of 3D high-dose lattice radiotherapy (LRT). International Journal of Radiation Oncology, Biology, Physics..

[9] Snyder AR (2004). Review of radiation-induced bystander effects. Hum Exp Toxicol.

[10] Garcia-Barros M, Paris F, Cordon-Cardo C (2003). Tumor response to radiotherapy regulated by endothelial cell apoptosis. Science.

[11] Kanagavelu S, Gupta S, Wu X (2014). In vivo effects of lattice radiation therapy on local and distant lung cancer: potential role of immunomodulation. Radiat Res.

[12] Sura S, Yorke E, Jackson A, Rosenzweig KE (2007). High-dose radiotherapy for the treatment of inoperable non-small cell lung cancer. Cancer J.

[13] Blanco Suarez JM, Amendola BE, Perez N, Amendola M, Wu X (2015). The use of lattice radiation therapy (LRT) in the treatment of bulky tumors: a case report of a large metastatic mixed mullerian ovarian tumor. Cureus.

[14] Amendola BE, Perez NC, Wu X, Amendola MA, Qureshi IZ (2019). Safety and efficacy of lattice radiotherapy in voluminous non-small cell lung cancer. Cureus.

[15] Pollack A, Chinea FM, Bossart E (2020). Phase I trial of MRI-guided prostate cancer lattice extreme ablative dose (LEAD) boost radiation therapy. Int J Radiat Oncol Biol Phys.

[16] Duriseti S, Kavanaugh J, Goddu S (2021). Spatially fractionated stereotactic body radiation therapy (lattice) for large tumors. Adv Radiat Oncol.

[17] Duriseti S, Kavanaugh JA, Szymanski J (2022). LITE SABR M1: a phase I trial of lattice stereotactic body radiotherapy for large tumors. Radiother Oncol.

